# What is the link between personality and food behavior?

**DOI:** 10.1016/j.crfs.2021.12.001

**Published:** 2021-12-08

**Authors:** Charles Spence

**Affiliations:** Oxford University, UK

**Keywords:** Personality, Taste, Gustation, Olfaction, Neophilia, Chile

## Abstract

A number of personality characteristics have been linked to various aspects of taste (gustation), trigeminal, and olfactory perception. In particular, personality traits have been linked to olfactory sensory thresholds and olfactory identification abilities, as well as to the sensory-discriminative aspects of taste/flavour perception. To date, much of the research in this area has focused on Sensation Seeking (including Experience Seeking, and Openness to Novel Experiences), with the latter being linked to a preference for spicy, and possibly also crunchy, sour, and bitter foods/drinks. Novelty-seeking has also been linked to a preference for salty foods, while anxious individuals appear to enjoy a much narrower range of foods. A bidirectional link has also been documented between taste and mood. Certain of the personality-based differences in taste/flavour perception and food behaviour have been linked to differences in circulating levels of neurotransmitters and hormones in both normal and clinical populations. Taken together, therefore, the evidence that has been published to date supports a number of intriguing connections between personality traits and taste perception/food behaviour.

## Highlights

• Personality characteristics linked to various aspects of taste (gustation), and trigeminal, perception.
• Personality traits also linked to olfactory thresholds and identification abilities.
• Food research has focused on Sensation Seeking and Openness to Novel Experiences.
• Novelty-seeking has also been linked to a preference for salty foods.
• Anxious individuals tend to enjoy a much narrower range of foods.

## 1 Introduction

The suggestion that differing taste preferences can be matched to one's personality often crops-up in marketing-led stories in the popular press, for some reason commonly linked to ice-cream (Anon., 2017; Atherley, 2021; see also Hawker & Monaghan, n.d.). Just take this from Atherley's recent press story: “CHOCOLATE ice cream lovers tend to be flirty and sensual — while vanilla fans are pure, a flavour expert suggests. Food psychologist Greg Tucker reckons which type we plump for reveals key personality traits.” Meanwhile, the personality types outlined in another blogpost include: The Optimist; The Realist; The Eccentric; The Ambitious; The Practical; and The Free Spirit.^1^ But is there any truth to the suggestion that there is a meaningful link between your personality and your taste preferences? The famous French gastronome Anton Brillat-Savarin would certainly seem to have thought so, with his much-quoted aphorism: “Tell me what you eat and I'll tell you who you are” (Brillat-Savarin, 1835/1884). At the same time, however, people also appear to find it surprisingly easy to ascribe personality characteristics to everyday food brands (e.g., such as Heinz soup or Oxo stock cubes; see MacClancy, 1992, p. 19), while one Australian wine brand has even gone so far as to label its wines with personalities, such as The Pugilist, The Opportunist, etc.

It turns out that there are, in fact, several neuroscience-based explanations for why one might expect there be a link between personality and taste/flavour preferences. However, at the outset, it is important to stress that the connection has more to do with the taste-buds (i.e., gustation), and the mouthfeel/trigeminal elements of tasting food (i.e., the burn of chile pepper) than with the olfactory contributions to flavour, important though the latter undoubtedly are (Spence, 2015). While there are some important individual differences in terms of our ability to smell the many different volatiles that contribute to flavour (e.g., see Blakeslee, 1935; Blakeslee and Salmon, 1931; Reed and Knaapila, 2010), these genetic differences have not, as yet, been linked directly to characteristic personality traits. So, for example, currently there are no known personality traits associated with a person's propensity to taste cilantro/coriander leaf as either citrusy/herbal or soapy and unpleasant, say (e.g., Eriksson et al., 2012; Mauer and El-Sohemy, 2012; McGee, 2010), nor with any of the many selective anosmias that have been reported in the literature over the years (Spence, 2017b).

By contrast, researchers have established a number of robust links between personality characteristics and our preferences for, and sensitivity to, basic tastes such as sweet, bitter, salty, sour, and the mysterious fifth taste of umami (Cecchini et al., 2019; Ikeda, 2002; Tracy, 2018). Furthermore, in the West at least, an individual's liking for the fiery heat of chile pepper has also been linked to their personality (see Spence, 2018b, for a review), and has been shown to correlate with levels of salivary testosterone (Bègue et al., 2015).

The link between basic tastes and personality is, of course, also firmly embedded in the English language in phrases such as ‘they are such a sweet person’ (Meier et al., 2012),^2^ ‘he is so bitter’, or ‘she was very sharp with me’.^3^ Just think about it, you would never describe someone as having a ‘grapefruit’ or ‘chocolate’ personality, now, would you?^4^ But if you were to describe them as sweet, then you would presumably be taken to mean that they are pleasant, kind, and gentle towards others. Intriguingly, tasting something sweet has also been shown to influence people's romantic perceptions too (see Ren et al., 2015). For instance, Ren and colleagues demonstrated that a hypothetical, if not an actual, relationship was evaluated more favourably by participants when they had been exposed to the sweet taste of Oreo cookies or a Fanta-like soft drink as compared to a non-sweet taste control (either salt & vinegar crisps or distilled water, depending on the study). Meanwhile, the participants in a third experiment expressed greater interest in initiating a relationship with a potential partner after they had been given something sweet to taste. Such results and observations hint, therefore, at the potentially close link that sometimes exists between personality and taste.

## 2 Individual differences in (taste) perception

It has long been known that we live in different worlds of taste (e.g., Bartoshuk, 1980; Blakeslee and Fox, 1932), with one of the most extensively-studied of differences relating to an individual's taster status. According to many decades of sensory science research, the population divides fairly evenly into three groups: supertasters, medium-tasters, and non-tasters (though see also Delwiche et al., 2001; Garneau et al., 2014; Lugg, 1966). Supertasters are much more likely to find foods such as Brussels sprouts and coffee very bitter (Keller et al., 2002; Spence, 2013). In the laboratory, a person's taster status is typically assessed by giving them a filter paper that has been soaked in a bitter-tasting chemical such as 6-*n*-propylthiouracil (PROP) or phenylthiocarbamide (PTC)^5^: These strips tend to taste very bitter (like a crushed aspirin) to supertasters, while non-tasters taste nothing at all. The genetic basis for taster status has been traced to alleles of the human TAS2R38 gene (Bufe et al., 2005; Hayes et al., 2008; Timpson et al., 2007). Supertasters also exhibit an enhanced response to the other basic tastes (Bartoshuk, 2000), and, what is more, they experience more oral-somatosensory texture from foods too (Eldeghaidy et al., 2011). It has even been suggested that supertasters may need less fat in their salad dressing etc. to get the same mouthfeel sensation as a non-taster (Tepper and Nurse, 1997).

According to Fischer et al. (1961), supertasters exhibit more food dislikes than non-tasters. Many years ago now, Corlis et al. (1967, p. 92) claimed that non-tasters are “factual down-to-earth organizers”, whereas supertasters are “theoreticians with insight who follow their inspirations”. This conclusion was reached on the basis of the responses given by 12 non-tasters and 10 supertasters (who fell at either end of a taste, i.e., gustatory, acuity continuum in a sample of 55 college students) to a series of Myers-Briggs type personality tests.^6^ There is some evidence to suggest that supertasters and tasters are also more apprehensive, tense, and imaginative than non-tasters (the latter considered to be placid, relaxed, and practical) on personality inventories (Mascie-Taylor et al., 1983).^7^ Those individuals who like bitter-tasting foods which, as we have just seen, is associated with taster status, also correlates with psychopathic tendencies. Or, as the authors of one study put it: ‘General bitter taste preferences emerged as a robust predictor for Machiavellianism, psychopathy, narcissism and everyday sadism.’ (Sagioglou and Greitemeyer, 2016; Sims, 2015).^8^

Another important individual difference as far as our worlds of taste is concerned relates to the liking for sweetness. Here the research shows that the population can be divided into three groups: sweet-likers, sweet-neutral, and sweet dislikers depending on their response to sugar as the concentration increases (e.g., Iatridi et al., 2019; Kim et al., 2014; Yang et al., 2019).^9^ And, once again, a genetic basis for, or contribution to, sweet-liking has now been identified, with a locus for this particular trait on chromosome 16 (see Keskitalo et al., 2007). Potentially relevant here, research by Meier et al. (2012) has shown that people with a so-called ‘sweet’ personality are more likely to like sweet-tasting foods. The latter researchers conducted a couple of experiments showing that individual differences in the preference for sweet foods predicted prosocial personalities, intentions, and behaviours. What is more, these researchers also found that we tend to believe that those strangers who like sweet food (candy) will be more likely to be agreeable as well.

### 2.1 Sensotypes: Different sensory preferences

Another potentially relevant individual difference to consider here relates to the concept of ‘sensotype’: This is the term that the anthropologist Mallory Wober (1966, 1991) first put forward some decades ago to describe the way in which different populations might differ in how much they value the information provided by each of their senses (see also Howes, 1991). While the term itself never really caught on in the academic literature, it is still interesting to note how marketers often want to draw a distinction between those customers who are high versus low in their ‘need for touch’ (e.g., Peck and Childers, 2003a, b; Peck and Wiggins Johnson, 2011). Those in the former category always want to feel the fabric before they buy their new outfit; they may also put greater store in the weight of the wine bottle when judging its quality (Piqueras-Fiszman and Spence, 2012). And, at least according to Gallace and Spence (2014), what is true for the skin on the outside of our bodies may well also be true when it comes to the oral-somatosensory experience of the mouthfeel and texture of foods in the oral cavity too. After all, think here only about how, as we have seen already, supertasters tend to experience more texture/mouthfeel from oily fatty foods than do non-tasters (Eldeghaidy et al., 2011). Put that together with the fact that food texture appears to be one of the key drivers of our food avoidance behaviours (as when people refuse to eat mushrooms, bananas, or oysters because of the ‘funny’ texture, or who find the seeds in kiwis or tomatoes off-putting; see Prescott, 2012, for a review), and a distinction between food texture/oral-somatosensation lovers and haters does not seem totally implausible.^10^

Broadening out the discussion, the study of other cultures soon reveals that there are those who have a much more developed vocabulary when it comes to describing smells (e.g., Majid, 2021; Majid and Burenhult, 2014). At the same time, we probably all know someone who really cares about how their food looks, and who is always loading pictures of their latest meals onto Instagram (see Spence et al., 2016, for a review). At the opposite extreme, one might also consider misophones: These are the unfortunate individuals who typically hate the sounds other people make when they eat and drink (Parker-Pope, 2011; Schröder et al., 2013; Spence, 2017b). Furthermore, elsewhere in psychology, there has also been a growing awareness that different people show different patterns of sensory preference/dominance (something that is also captured as different learning styles in Neuro-Linguistic Programming; e.g., Craft, 2010). Hence, while there is currently undoubtedly still a paucity of evidence concerning the idea that consumers can be fruitfully classed as having different ‘food sensotypes’, this still feels like an intriguing area for future research, especially for those interested in the topic of multisensory perception.

## 3 Personality traits and their relevance to taste (gustation)

According to the theory of the Big Five personality traits (e.g., Costa and McCrae, 1992; Matthews et al., 2003; McCrae and Costa, 2008), five key factors determine a person's personality namely: extraversion (outgoing/energetic vs. solitary/reserved); agreeableness (friendly/compassionate vs. critical/rational); openness to experience (inventive/curious vs. consistent/cautious); conscientiousness (efficient/organized vs. extravagant/careless); and neuroticism (sensitive/nervous vs. resilient/confident). Intriguingly, several of these traits correlate with various aspects of people's food behaviour. At the same time, however, it is also worth noting here how an individual's long-term personality traits are likely going to be linked to their short-term emotional state (e.g., Diener and Larsen, 1984; Eid and Diener, 1999; see Section 4).

One of the most frequently-mentioned aspects of personality relates to the dimension of extraversion/introversion (Cain, 2012). Extraverts tend to like more sensation, whether from the food they eat, or from the music that they listen to. Extraverts also tend to engage in more sensation-seeking behaviours than do introverts (Zuckerman, 1979; Zuckerman and Bone, 1972; Zuckerman et al., 1980). In 2007, occupational therapist, Winnie Dunn, attempted to classify people into one of four sensory types: Seekers, Bystanders, Avoiders, and Sensors (Dunn, 2007). These labels, which appear to broadly align with a number of the personality traits that have just been mentioned have also been explicitly linked to people's food preferences. For instance, one of the reviews of Dunn's book that appeared in the popular press, asked: “Are you a sensory junkie? How do you respond to bright lights, soft clothes, loud music and spicy food? …. Some people adore the feel of silk and velvet. They probably also savour the flavour of fresh peach, dress brightly and crave company.” (Rix, 2007). The text goes on to suggest that ‘seekers’ like spicy food, whereas ‘avoiders’ make much narrower food choices. This sounds very much like sensation-seeking and neophobic food behaviours, respectively. Dunn herself, though, suggests that as individuals we may exhibit behaviours that are consistent with several of these sensory types at different times, or in different aspects of our everyday behaviour. It is perhaps for this very reason that the classificatory scheme introduced by Dunn has seemingly mostly been ignored by the scientific community in the years since it was first put forward.

Many years ago now, Wolowitz (1964) drew a distinction between bland, sweet, and soft foods on the one hand and spicy, sour, and crunchy foods on the other, suggesting that sensation seekers would, if anything, tend to prefer the former (Kish, 1970; Kish and Donnenwerth, 1972). That said, although a significant correlation between sensation seeking and spicy, sour, and crunchy food was documented, it was very modest. Similarly, Brown et al. (1974) also reported a weak, but significant, correlation between scores on Garlington and Shimota (1964) Change Seeker Index and the preference for spicy food (as well as with watching X-rated movies and gambling). Elsewhere, Back and Glasgow (1981) found that self-proclaimed gourmets scored significantly higher than vegetarians on measures of the general Sensation Seeking Scale (SSS), as well as the Experience Seeking subscale.^11^ Otis (1984) documented a modest correlation between scores on the Experience Seeking subscale of the SSS and an individual's willingness to taste various foods, consistent with previous findings suggesting a weak relationship between food preferences and novelty seeking (Brown et al., 1974; Kish and Donnenwerth, 1972). In a survey of more than 300 people ranging in age between 14 and 68 years, Logue and Smith (1986, p. 109) noted that: “Preferences for spicy foods or foods likely to cause illness were positively correlated with sensation seeking while preferences for sweet or bland foods or foods unlikely to cause illness were negatively correlated with sensation seeking.”^12^ Interestingly, however, Logue and Smith claimed not to find any link between food texture and sensation-seeking.

At this point, it is perhaps worth highlighting the fact that the majority of studies that have attempted to examine the relationship between personality traits with taste preferences have been conducted on self-rated food preferences rather than on actual preferences collected with real foods (i.e., rather than merely with written food descriptions).^13^ Otis (1984) study is unusual in this regard inasmuch as bite-sized pieces of 12 actual foods were placed in front of participants: octopus, hearts of palm, seaweed, soya bean milk, blood sausage, Chinese sweet rice cake, pickled watermelon rind, raw fish, quail egg, star fruit, sheep milk cheese, and black beans. Otis found that an individual's level of state anxiety was correlated with their willingness to try foods. More specifically, the most anxious individuals were the least willing when it came to trying novel foods (see also Smith, Powell and Ross, 1955a, b). This result was taken to suggest that food novelty may be important in terms of the correlation between an individual's food-preferences and their sensation-seeking behaviour. Such findings, note, fit with earlier observations suggesting that neurotically anxious individuals tend to have a greater number of food aversions than normal (Gough, 1946; Wallen, 1945). Otis (1984, p. 743) wound-up concluding that: “In light of the relatively low correlations between the general personality measures and willingness to try new foods, this result suggest that patterns of food choice may be quite independent of general preferences and responses in other areas of life (Rozin and Schiller, 1980).” Note that Otis based this conclusion on the fact that the willingness to taste unusual foods appeared largely unrelated to preferences for engaging in other kinds of novel, or risky, activity.

Terasaki and Imada (1988) also highlighted a link between sensation seeking and food preferences. These Japanese researchers conducted a study in which 105 students completed both the SSS and a food preference questionnaire.^14^ Those who scored high on the SSS were more likely to report liking spicy foods, meats, and alcoholic beverages (see also Logue and Smith, 1986). A significant positive correlation was also detected between a participant's liking for spicy food and the Thrill and Adventure Seeking, and Experience Seeking, sub-components of the SSS (at least in this Japanese sample). Meanwhile, in a conference abstract, Day et al. (2008) reported that those participants who were high in ‘novelty seeking’ exhibited a strong preference for salty tastes, whereas those who were high in ‘reward dependence’ show a strong preference for sweet tastes instead. (These were described as temperamental personality dimensions.) It has also been suggested that ‘sensation seeking’ may play a significant role in determining a person's intake of caffeine (Mattes, 1994), as well as their consumption of products such as coffee, tea, and chocolate (Evans et al., 2006). Furthermore, sensation-seeking has also been tied to an individual's liking and intake of chile (Byrnes & Hayes, 2013, 2015; Rozin and Schiller, 1980; Stevens, 1990). Furthermore, as has been mentioned already, salivary testosterone levels correlate with how much spice (Tabasco) people chose to add to their food in a laboratory setting (Bègue et al., 2015).

According to one popular suggestion, it is the endorphin hit, much like running a marathon, that people come to associate with the consumption of increasingly-spicy food that they, in some sense, crave or even become ‘addicted’ to (Rozin and Schiller, 1980; see also Byrnes and Hayes, 2015, 2016), due to the release of endogenous opioids (Rozin, 1987). It is, though, important to note that the long-time food researcher, Paul Rozin's, masochistic/thrill-seeking account of chilli consumption makes more sense in those countries such as the USA and the UK, where an ability to handle the burning heat has, at least amongst a certain section of society, become associated with outward signs of masculinity (cf. Cross et al., 2013). What is more, while the hypothesis has been known to make its way into the popular press (Gorman, 2010; see also MacClancy, 1992, p. 21), convincing empirical support for the hypothesis still has not been forthcoming nearly four decades after the suggestion first appeared in print (see Spence, 2018b, for a review).^15^

There have also been several reports of certain other personality attributes, such as, for example, the openness to experience, correlating with particular food behaviours. For instance, in one study, those participants who scored above average on openness (that is, the preference for new experiences and variety) ate about 4.5 more servings of combined fruit and vegetables per week than their peers who were less open; They also consumed less unhealthy food, such as potato chips or fries (Conner et al., 2017). In an interview, the study's lead author Tamlin Conner, a professor at the University of Otago in New Zealand, told *The Huffington Post* that: “It's likely that people who are open to new experiences and crave variety extend those same attitudes toward food, …their personality may make them more eager to try new fruits and veggies, or brave enough to keep experimenting with ones they don't initially like.” (quoted in Strutner, 2017).

A separate line of individual differences research in the food psychology literature has focused on food neophobia/neophilia (e.g., Arvola et al., 1999; Henriques et al., 2009; Pliner and Hobden, 1992; Veeck, 2010). The population can be divided into those who do not like trying new foods and others who are much more open to novel food experiences. Notice how this would appear to be a food-specific form of the openness to new experiences. Food neophiles are classed as adventurous eaters for those foods that are considered new or different (Latimer et al., 2015).^16^ Latimer and colleagues found that food neophiles tended to have a lower Body Mass Index (BMI), were more likely to cook in order to connect to their heritage, and also tended to be more concerned about the healthfulness of the food they eat. Elsewhere, researchers have also documented a relationship between food neophobia and vegetable consumption in children (Knaapila et al., 2011; cf. de Bruijn et al., 2005; Keller et al., 2014; Vollrath et al., 2012).

### 3.1 Personality influences on olfactory perception

By contrast to the relatively large literature linking taste (gustation) to personality, those studies that have examined the link between personality and the sense of smell (i.e., olfaction) is much smaller. That being said, what research there is suggests a tentative link between personality variables and olfactory sensitivity (Koelega, 1994; Pause et al., 1998), and with odour identification abilities (Larsson et al., 2000; though see also Hvastja and Zanuttini, 1991). For instance, Rovee et al. (1973) reported that highly-anxious women exhibited reliably higher thresholds for octanol than did those women who were low in anxiety. Meanwhile, the neurotic and anxious individuals in a sample of 75 young adults studied by Chen and Dalton (2005) were selectively biased toward affective rather than neutral odorants. In particular, those women who scored high in trait anxiety perceived emotionally valenced odorants (pleasant citrus and unpleasant fecal scents) as stronger (in terms of their perceived intensity) than the neutral one (rubbing alcohol), while those male participants who scored high in neuroticism or anxiety detected the pleasant and unpleasant odorants somewhat faster than the neutral smell. Shyness has also been linked to olfactory perceptual thresholds (Herbener et al., 1989), with extremely shy males having significantly lower olfactory thresholds (meaning that they are more sensitive to smells). By contrast, introversion-extraversion is not a strong predictor of olfactory performance (Chen and Dalton, 2005; Filsinger et al., 1987; Koelega, 1970, 1994; Larsson et al., 2000),^17^ with Pause et al. (1998) reporting that neuroticism was a stronger predictor for olfactory sensitivity than extraversion.

The results of a study of a group of 532 participants aged 45–87 years from the Swedish Adoption/Twin Study of Aging assessed several personality traits revealed that odour identification abilities were predicted by neuroticism, impulsivity, and a lack of assertiveness (Larsson et al., 2000). In particular, a negative correlation was documented between impulsivity and lack of assertiveness (meaning that those who were high in impulsivity and lacking assertiveness performed poorly on olfactory identification), while there was a positive correlation for openness. In this case, the suggestion from the authors was that impulsive individuals may simply have been negligent on task whereas those lacking assertiveness may be indecisive in answering (note how this might be especially problematic in the case of an olfactory identification task). It is important to stress, though, how no clear distinction has been made between food-related and food-unrelated odours on the one hand, nor between orthonasal and retronasal olfactory perception on the other in the majority of these studies. Taken together, therefore, while it is undoubtedly the case that olfactory perceptual abilities (at both the threshold and suprathreshold levels) can be influenced by personality variables, the literature on personality and olfaction has not given rise to any specific predictions about the likely food preferences as a function of the personality type.

### 3.2 Interim summary

Put all of the research together and it starts to become increasingly clear just how many of our food preferences as adults may actually be linked to aspects of our personality (Spence, 2017a. As we have seen already, sensation-seekers tend to like it spicy (and possibly also sour and crunchy; e.g., Kish, 1970; Logue and Smith, 1986; Wolowitz, 1964). Meanwhile, at least according to the results of one study by Day et al. (2008), novelty-seekers show an enhanced liking for salty foods too. And according to the latest research, personality traits can also be tied to liking and intake of pale ale style beers (Higgins et al., 2020). In particular, Higgins et al. found that high sensation seekers reported an increased liking of bitter pale ale (at least in those who also perceived the bitterness of quinine to be high). Intriguingly, pale ale drinkers have been classed as novelty seekers (Malone and Lusk, 2018). I would therefore like to suggest, contrary to the claims put forward by Otis (1984), that those individuals who show an openness toward trying new taste experiences/flavour combinations, and who are intrigued by the latest fusion foods (see Spence, 2018a), will likely also show an openness to other kinds of aesthetic experience as well (cf. Eysenck, 1940).

Those who are open to new experiences have been shown to like a wider arrange of foods, whereas those who are anxious tend to exhibit a reduced range of food likes (neophilic and neophilic, respectively). Olfactory threshold and olfactory identification abilities have also been linked to various personality traits but again, without any very specific predictions concerning likely food preferences (Chen and Dalton, 2005; Larsson et al., 2000; Pause et al., 1998; Filsinger et al., 1987). At the same time, however, it should be stressed that one of the challenges with reviewing the literature on personality and taste relates to the wide range of different personality scales that have been used by researchers over the years. These include the Myers-Briggs type personality scales (Myers, 1962) by Corlis et al. (1967); Cattell's Sixteen Personality Factor Questionnaire (Cattell and Eber, 1972) by Mascie-Taylor et al. (1983); The Maudsley Personality Inventory (Eysenck, 1959) by Koelega (1994), etc. While a number of the dimensions, such as introversion-extraversion tend to appear across different measurement instruments, others are simply not synonymous. Another relevant factor to bear in mind is how what counts as a novel food differs by culture/country and also changes substantially as the decades go by. This obviously makes it that much harder to generalize about the nature of any specific foods, or food groups, that are more popular amongst e.g., novelty seekers/those who are high in their openness to new experiences, for example.

## 4 Food and behaviour: The bidirectional relationship between mood and taste

The relationship between personality and taste operates bidirectionally: That is, while certain emotional states can affect our taste perception, experiencing particular tastes can also make us more likely to behave in certain predictable ways too (Meier et al., 2012). Indeed, the very act of eating has been shown to affect our mood as well as the decisions that we make. What is more, these decisions sometimes have serious consequences as in the case of the Israeli judges who were shown to be much more likely to grant parole straight after a meal break than at the end of a session (Danziger, Levav and Avnaim-Pesso, 2011a, b). Experiencing different tastes has, across a number of studies, been shown to affect people's mood, their perception, and their behaviour. So, for example, experiencing a sour-taste has been shown to make people more likely to take risks (Vi and Obrist, 2018), while tasting something sweet appears to make us more likely to believe that we have pro-social tendencies (Meier et al., 2012); It can also make us temporarily more romantic (Ren et al., 2015; Wang et al., 2019; Zaraska, 2015). According to Meier et al. (2012), savouring a sweet food leads to an increase in self-reports of agreeable and helping behaviour.^18^ Meanwhile, according to Chan et al. (2013), love proved to be a good metaphor for sweetness, whereas people associated jealousy with bitterness and sourness. Getting people to remember an episode of romantic love resulted in their rating three foods (a sweet-sour pastille, a bitter-sweet chocolate, and tasteless water) as tasting sweeter than those who had recalled a jealous love, neutral or happy memory instead. Intriguingly, the effects of love on sweetness rating were obtained regardless of what the food was, and regardless of whether the food was sweet to begin with.

Sagioglou and Greitemeyer (2014) reported that experiencing a bitter-tasting drink made people more hostile. Meanwhile, Eskine and colleagues (Eskine et al., 2011) reported that people tend to make harsher judgments (of moral transgressions) when experiencing a bad taste in the mouth (than a sweet tasting food or water). The claim here being that the physical disgust elicited by tasting something bitter elicited feelings of moral disgust.^19^ Certain experiences have also been shown to lead to a bad taste in the mouth (Eskine et al., 2011; Eskine et al., 2012; see also Bratanova et al., 2015). Having people remember being treated unfairly at work evokes a feeling of disgust which can then lead to bitter tastes being rated as more intense (Skarlicki et al., 2013). PROP taste sensitivity has also been related to visceral but not moral disgust (Herz, 2011). That said, a recent multi-lab attempt to replicate Eskine et al. (2011) findings concluded that the evidence was more consistent with a null effect than anything else (see Ghelfi et al., 2020). Taken together, such findings, at least the replicable ones (!), obviously have relevance to those working in the field of gastrodiplomacy (see Spence, 2016, for a review; see also Woolley and Fishbach, 2017).

There is plenty of research out there showing how people's rating of the taste of different foods, or model solutions, may change predictably as a result of laboratory-induced (Macht, 1999; Macht and Mueller, 2007a; Macht et al., 2002; Wang and Spence, 2018), or naturalistic (Noel and Dando, 2015), changes in mood. For instance, according to research from Noel and Dando, those fans whose ice hockey team had just won the game they were watching rated a lemon-lime sorbet as tasting significantly sweeter than those who had been supporting the loosing team instead. At the same time, however, increased levels of anxiety, as induced in lab-rats by the presentation of unpredictable loud noises, results in sweetness becoming more pleasant (Kupfermann, 1964). The suggestion here being that we may be drawn toward those tastes that signal the energy we might need to help us get out of an anxiety-causing situation (see Spence, 2014, for a review). At the same time, however, stress situations have also been reported to make both rats and humans more sensitive to bitterness (Dess, 1992; Dess and Edelheit, 1998).

Macht and colleagues conducted a number of studies in which they induced either a positive or negative mood in their participants using pre-selected short film clips, before assessing the impact on taste ratings (Macht and Mueller, 2007a). Macht, Roth, and Ellgring (2002) reported that watching a joyful clip (from ‘When Harry met Sally’) chocolate taste more pleasant too. Similarly, Greimel et al. (2006) reported that people rate a sweet drink as tasting more pleasant after watching a joy-inducing than a sad movie clip (see also Platte et al., 2013). Macht and Mueller (2007b) assessed the PROP taster status of 108 individuals and experimentally induced different mood states in the laboratory using emotional film clips. Valence ratings were taken before and after. Intriguingly, the PROP-tasters reported more intense emotions, increased anger tension and fear when watching angry film clips than did the non-tasters. By contrast, no difference between the taster groups were observed in terms of the ratings of sad movie clips. Elsewhere, meanwhile, other researchers have shown that PROP taster status and self-perceived food adventurousness influence food preferences (Ullrich, Touger-Decker, O'Sullivan-Maillet and Tepper, 2004).

According to Macht (1999), people tend to associate joy with increased appetite, while they associate sadness with a decreased appetite. Macht et al. (2002) found that people reported more hunger after watching an anger- or joy-inducing movie clip than after watching a fear or sadness-inducing clip. Lyman (1982) suggested that positive emotions are associated with an increase in the consumption of healthy foods, whereas negative emotions are associated a greater consumption of junk food instead.

### 4.1 Clinical mood disorders and taste perception

It has long been known that taste thresholds can change significantly in those who are suffering from clinically-relevant mood disorders such as depression (Miller and Naylor, 1989; see also Joiner et al., 2004; Thomas et al., 2014). Indeed, severely depressed patients are less sensitive to all tastes, especially sweet (Steiner et al., 1969) with this deficit typically normalizing on recovery (cf. Arbisi et al., 1996). Depressed individuals have also been reported to give lower intensity responses to suprathreshold solutions of sucrose (Amsterdam et al., 1987; see also Willner and Healy, 1994). Intriguingly, in clinical populations, it has been shown that there is a link between taster status and clinical depression (e.g., Whittemore, 1986, 1990; see also Dess and Edelheit, 1998). Whittemore (1986, 1990) documented longer and more severe depressive episodes for those with heightened taste sensitivity (tasters/supertasters) as well as higher rates of familial depressive illness, although it should be noted that this was only assessed this in a small group of females. In contrast, though, Joiner et al. (2004) found that supertasters appeared less likely to suffer from depression. According to research by Thomas et al. (2014), a diminished sensitivity to the taste of PTC is associated with general decrements in hedonic capacity (anhedonia).

Supertasting rats, i.e., those who find saccharin very bitter show different behaviours, specifically an increased emotional reactivity in response to negative stimuli (Dess and Minor, 1996). Dess (1992) reported that bitter sensitive rats were more easily stressed, and socially subordinate. Something very similar has also been reported in people. Supertasting people also tend to find saccharin very bitter (see Bartoshuk, 1979), while induced stress in normal people leads to an increased sensitivity to the bitter taste of saccharin (Dess and Edelheit, 1998). Supertasters are generally more reactive. According to research from Herbert et al. (2014), people's sensitivity to bitter taste (PROP solutions) modulates their emotional approach/avoidance behavior, as indexed by the affective startle paradigm (one of the classic paradigms from experimental psychology). In particular, PROP tasters showed facilitated response priming to emotional pictures – that is, they were more primed to approach/avoid tendencies. Macht (1999) has suggested that those individuals who are more sensitive to bitter tastes are also likely to jump/react more strongly to sudden loud noises.

Those suffering from panic disorder, by contrast, have been shown to exhibit a reduced sensitivity to quinine (DeMet, Stein, Tran, Chicz-DeMet et al., 1989). Meanwhile, levels of anxiety are positively correlated with an individual's bitter and salt taste thresholds (Heath et al., 2006). Potentially relevant here, human taste thresholds are affected by levels of circulating neurotransmitters such as serotonin and noradrenaline (Heath et al., 2006). In particular, Heath et al. (2006) reported that enhancing serotonin reduced sucrose (sweet) and quinine (bitter) thresholds significantly (by 27% and 53%, respectively), while enhancing noradrenaline significantly reduced bitter and sour taste thresholds (by 39% and 22%, respectively). Given the finding that the general level of anxiety is directly related to taste perception, the altered taste and appetite that is sometimes seen in affective disorders may reflect an actual change in the gustatory system.

Finally, here, it is worth noting how there may also be a link here between the stereotypical facial expressions that are associated with experiencing the different basic tastes (see Steiner, 1974, 1979; Steiner et al., 2001; Spence, 2012; Weiland et al., 2010) and those that are associated with displays of facial emotion (e.g., Liang et al., 2021; cf. de Wijk et al., 2021; Greimel et al., 2006).^20^ This connection certainly makes sense in terms of the increasingly-popular theories of embodied cognition (see Wilson, 2002). Very recently, in fact, Liang et al. (2021) demonstrated that tasting food (sweet vs. acidic) affected people's ability to identify/recognize the facial emotion of others. Negative faces were identified significantly faster with an acidic taste in the mouth rather than with a sweet taste.

### 4.2 Interim summary

Taken together, the diverse bodies of research that have been briefly summarized in this section support the claim that there may be multiple routes/mechanisms by which personality traits and taste preferences and food behaviours might be linked. Once again, though, it is worth stressing how it is the basic taste qualities, especially bitter, sweet, and to a lesser extent salty and sour that are both associated with personality characteristics and mood disorders, as well as biasing our mood/thought patterns when we experience a certain basic taste quality. In clinical populations, there would appear to be a connection between the role played by various neurotransmitters on mood/personality and on gustatory sensory thresholds (Heath et al., 2006; Herbener et al., 1989). High levels of central norepinephrine have been hypothesized to influence both shyness and olfactory sensitivity (Herbener et al., 1989). Furthermore, sex-linked hormones such as testosterone have also been linked to personality attributes and food behaviours as well (Bègue et al., 2015). A 2015 study in rodents revealed that the receptors for glucocorticoids, considered primary stress hormones, are located inside the taste buds that detect sweetness and the umami taste (Ogawa et al., 2015). According to one suggestion, if glucocorticoids flood the body when we are stressed this might inhibit the functioning of these classes of taste receptor, and hence altering an individual's responsiveness to the associated tastants at either the threshold and/or suprathreshold levels (see Zaraska, 2015). At the same time, however, it should also be borne in mind that we never really experience pure tastants in everyday life, and hence the predictions of much of this literature for our everyday food behaviours/experiences must remain unclear.

Taken together, therefore, the evidence published to date therefore supports a number of intriguing connections between personality traits and taste perception/food behaviour. Finally, here, the link between an individual's diet and their body odour should not be ignored either (see Havlicek and Lenochova, 2006; Zuniga et al., 2017).

## 5 Conclusions

In conclusion, the research that has been reviewed here highlights how a number of personality characteristics have been linked to various aspects of taste (gustation), trigeminal, and olfactory perception, and diet (Keller and Siegrist, 2015; Kikuchi and Watanabe, 2000). Particular personality traits have been linked to olfactory sensory thresholds and olfactory identification abilities, as well as to the sensory-discriminative aspects of taste/flavour perception. To date, much of the research in this area has focused on Sensation Seeking (including Experience Seeking, and Openness to Novel Experiences), with the latter being linked to a preference for spicy, and possibly also crunchy, sour, and bitter foods/drinks. Novelty-seeking has also been linked to a preference for salty foods. Anxious individuals tend to enjoy a much narrower range of foods. There is presumably also a link here to food neophobia/neophilia. A bidirectional link has also been documented between taste and mood.

Intriguingly, certain of the personality-based differences in taste/flavour perception and food behaviour have been linked to differences in circulating levels of neurotransmitters and hormones in both normal and clinical populations. Taken together, therefore, the evidence published to date supports a number of intriguing connections between personality traits and taste perception/food behaviour. At the same time, however, making specific food (or food group) preferences linked to particular personality traits is made all the more difficult by the variety of different measurement tools that have been used to assess personality traits over the decades and the fact that the foods that are popular/unusual has also changed over the decades and also varies as a function of culture.

## Research funding

This research did not receive any external funding.

## CRediT authorship contribution statement

**Charles Spence:** The author confirms that he wrote all parts of the review.

## Declaration of competing interest

The authors declare that they have no known competing financial interests or personal relationships that could have appeared to influence the work reported in this paper.

## Footnotes

1 According to Juliet A. Boghossian, a behavioural food expert, an individual's personality traits can be identified simply based on how a person chooses to eat their ice-cream. According to the blog-post: “people who prefer their ice cream on a cone are more often to be optimists, while those who enjoy their scoop in a cup are more often realists.” (Anon, 2017).

2 People are sometimes also described as ‘sweetie’, honey’, or ‘sugar’ (Ren et al., 2015). In fact, the expression ‘love is sweet’, or its equivalent, can be found in many different languages, including both German and Mandarin (see also Chan et al., 2013).

3 Though, as one person asked on a Reddit thread a few years ago: “Food can be sweet, salty, sour, and bitter. People's personalities can also be sweet, salty, sour, and bitter. I wonder what an umami personality is like.” (https://www.reddit.com/r/Showerthoughts/comments/6te8tg/food_can_be_sweet_salty_sour_and_bitter_peoples/).

4 Though, of course, we sometimes say that something ‘smells fishy’ is we are suspicious (see Lee and Schwarz, 2012). And, as MacClancy (1992, p. 2) notes: “We use food words as metaphors, as ways of talking about something else: he's a nut; your car is a lemon; my daughter is the apple of my eye; and the man just blew you a raspberry.”

5 Note that PROP is mostly used these days given that PTC is potentially toxic.

6 The personality test assesses four dimensions: extroversion-introversion; sensation-intuition; thinking-feeling; and judging-perceiving.

7 Personality, in this case, was assessed using Cattell's Sixteen Personality Factor Questionnaire (Cattell and Eber, 1972) based on 141 first and second year Cambridge University undergraduates.

8 It is, though, important to stress here that correlation does not imply causation. That is, you are not necessarily a psychopath should you happen to be one of those people who happens to like bitter-tasting foods and drinks.

9 According to Iatridi et al. (2019), the sweet-liker phenotype is characterized by a rise in liking as sucrose concentration increases, an inverted U-shaped phenotype exhibits a maximum liking at around 0.25 M sucrose concentration, and a sweet-disliker phenotype characterized by a decline in liking as a function of sucrose concentration.

10 It would be interesting in future research to determine whether an individual's response on the well-established ‘need for touch’ scale is significantly associated with any particular aspects of their food behaviour (especially food neophobia).

11 Note that the four sub-scales of the SSS include, Thrill and Adventure Seeking, Experience Seeking, Disinhibition, and Boredom Susceptibility.

12 And if you were wondering which are the foods that are likely to cause illness, Logue and Smith (1986) had in mind alcohol and shellfish.

13 Though note that when the two have been compared, they have generally been found to align reasonably well, with the possible exception of bitterness (see Day et al., 2008).

14 Interesting cultural differences are also highlighted when one compares what counts as a novel/unusual food in these various studies. For instance, Terasaki and Imada (1988) included the following 11 really rather unusual foods in their list: locust, larva of the bee, bullfrog, snake, earthworm, slug, lizard, newt, gecko, snail and Japanese diving beetle (see also Peryam, 1963).

15 This is perhaps not so surprising when it is remembered that Rozin and Schiller (1980) themselves actually failed to demonstrate a significant correlation between chili preference and a selection of masochistic activities such as taking a very hot bath, watching sad movies, and taking part in dangerous sports.

16 This should perhaps not come as such a great surprise given that one of the questions in Zuckerman's SSS explicitly asks about food, namely “I like to try new foods that I have never tasted before” (Zuckerman et al., 1964).

17 This is surprising inasmuch as introverts have been reported to have higher sensitivity in other sense modalities (Eysenck and Eysenck, 1985).

18 Interestingly, oral stimulation with sucrose has been shown to increase levels of dopamine in the accumbens in rats (Hajnal et al., 2004).

19 Though, I would argue that while bitter tastes may be very unpleasant that does not, in-and-of-itself make them disgusting.

20 Perhaps captured by the expression ‘to pull a sour face’.
